# Food availability modulates temperature‐dependent effects on growth, reproduction, and survival in *Daphnia magna*

**DOI:** 10.1002/ece3.5925

**Published:** 2019-12-27

**Authors:** Gustavo S. Betini, Xueqi Wang, Tal Avgar, Matthew M. Guzzo, John M. Fryxell

**Affiliations:** ^1^ Department of Integrative Biology University of Guelph Guelph ON Canada; ^2^ Department of Wildland Resources Utah State University Logan UT USA

**Keywords:** *Chlorella vulgaris*, climate change, *Daphnia magna*, demographic rates, life history, lifetime reproductive success, metabolism, pace of life, temperature‐size rule, vital rates

## Abstract

Reduced body size and accelerated life cycle due to warming are considered major ecological responses to climate change with fitness costs at the individual level. Surprisingly, we know little about how relevant ecological factors can alter these life history trade‐offs and their consequences for individual fitness. Here, we show that food modulates temperature‐dependent effects on body size in the water flea *Daphnia magna* and interacts with temperature to affect life history parameters. We exposed 412 individuals to a factorial manipulation of food abundance and temperature, tracked each reproductive event, and took daily measurements of body size from each individual. High temperature caused a reduction in maximum body size in both food treatments, but this effect was mediated by food abundance, such that low food conditions resulted in a reduction of 20% in maximum body size, compared with a reduction of 4% under high food conditions. High temperature resulted in an accelerated life cycle, with pronounced fitness cost at low levels of food where only a few individuals produced a clutch. These results suggest that the mechanisms affecting the trade‐off between fast growth and final body size are food‐dependent, and that the combination of low levels of food and high temperature could potentially threaten viability of ectotherms.

## Graphical abstract

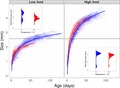

## 1 INTRODUCTION

Organisms are regularly exposed to several environmental stressors that can interact with each other to affect individual fitness. Predicting the magnitude and direction of these effects is difficult because different stressors can have additive, synergistic and even antagonistic effects (Coors & Meester, 2008; Crain, Kroeker, & Halpern, 2008; Galic, Sullivan, Grimm, & Forbes, 2018; Heugens, Hendriks, Dekker, Straalen, & Admiraal, 2001). For example, variation in food availability and temperature can either increase or decrease the toxicity of many substances (Boone & Bridges‐Britton, 2006; Heugens et al., 2001) and a recent meta‐analysis showed that, in freshwater organisms, responses to different stressors tend to be antagonistic or additive, in contrast with the prevalence of synergistic effects seen in marine systems (Jackson, Loewen, Vinebrooke, & Chimimba, 2016). Thus, it is essential that we assess the different ways by which organisms respond to multiple stressors.

Increases in average temperature, as well as in the magnitude and timing of temperature variation, are important ecological stressors related to climate change that affect all levels of biological organization. Warmer temperatures can advance reproductive maturation, increase reproduction frequency and shorten life span, leading to an accelerated life cycle (Bestion, Teyssier, Richard, Clobert, & Cote, 2015). These individual effects have been shown to scale up to destabilize population dynamics and increase the risk of extinction (Bestion et al., 2015). High temperature can also lead to a decline in mean body size of a given population (Atkinson & Sibly, 1997), which has been considered a general response to global warming, especially in ectotherms (Daufresne, Lengfellner, & Sommer, 2009; Ohlberger, 2013; Sheridan & Bickford, 2011). However, the effect of temperature on organismal fitness often depends on several other ecological factors. For example, many studies have shown that body size shrinkage is mediated by genetic background (Cambronero, Beasley, Kissane, & Orsini, 2018; Hoefnagel, Vries, Jongejans, & Verberk, 2018), pesticides (Cambronero et al., 2018), and food availability (Cambronero et al., 2018; Heugens et al., 2001; Orcutt & Porter, 1984).

Variation in food availability is particularly important because it can also influence final adult body size (Atkinson, 1994; Atkinson & Sibly, 1997; Kooijman & Kooijman, 2010; Vidal, 1980) and demographic parameters, with important consequences for the resilience of populations (Gardner, Peters, Kearney, Joseph, & Heinsohn, 2011; Hoy, Peterson, & Vucetich, 2017; Sheridan & Bickford, 2011; Yom‐Tov, Yom‐Tov, Wright, Thorne, & Feu, 2006). Organisms often experience wide seasonal variation in food availability, and climate change is altering the timing, amount and variation of many resources (Williams et al., 2017). For example, global warming can have a negative impact on consumer resource due to mismatches between seasonal resource peaks and consumer breeding phenology (Both et al., 2010; Cahill et al., 2012; Parmesan, 2006). In some systems, food is becoming more abundant (Cox, Betts, Jones, Spall, & Totterdell, 2000; Parton, Scurlock, Ojima, Schimel, & Hall, 1995) due to positive temperature‐dependent effects on primary productivity (Taucher & Oschlies, 2011), while many organisms are experiencing higher variation in food availability (Kraemer, Mehner, & Adrian, 2017; O’Reilly, Alin, Plisnier, Cohen, & McKee, 2003; O’Reilly et al., 2015GL). Although many studies have demonstrated significant interactions between temperature and food stress (Cambronero et al., 2018; Heugens et al., 2001; Jackson et al., 2016; Orcutt & Porter, 1984), it is not clear whether high levels of food abundance could compensate for the negative effects of high temperature on body size imposed by an accelerated pace of life (Gardner et al., 2011) or if low levels of food could inhibit an accelerated life cycle.

Here, we applied a factorial experimental design using the water flea *Daphnia magna* as a model system to disentangle the interactive effects of food and temperature on individual fitness. We exposed 412 *Daphnia magna* individuals to either high or low levels of food abundance as well as either high or low temperature and tracked reproduction, survival, and somatic growth over their entire lifespan. *Daphnia* is often promoted as an ideal model organism to investigate the effects of climate change (Scheffers et al., 2016). Like *Daphnia*, 99.9% of the species on Earth are ectothermic and their metabolic rates are expected to increase by 10%–75%, making them particularly vulnerable to global warming (Bickford, Howard, Ng, & Sheridan, 2010; Daufresne et al., 2009). Our dataset allowed us to investigate the combined effects of variation in temperature and/or food abundance on adult body size and several life demographic parameters, such as size and age at first reproduction, lifetime reproductive success, and life span.

## 2 METHODS

Females for this study originated from a clonal population of *Daphnia* that was first raised in two 26,000 L tanks for 18 months, such that all individuals experienced wide variation in food abundance caused by population fluctuations, but were exposed to either high or low levels of ambient temperature (Betini, Avgar, McCann, & Fryxell, 2017). After inoculation with algae and *Daphnia*, the tanks were not disturbed. Two tanks were first inoculated with a clonal population of *D. magna* that were kept in laboratory at 20°C with 12 hr:12 hr dark:light cycle. Females were collected from these two tanks whose mean temperature had been kept at either 15°C or 25°C (eight females from each tank), with temperature at different depths in the water column varying between 13 to 18°C (14.93°C, ±1.95; mean and *SD*) and 23 to 31°C (24.84°C, ± 3.10), respectively. Individuals from both tanks were brought to the laboratory and kept in incubators with constant temperature at either 15 or 25°C, according to the average temperature they experienced in the mesocosm tanks. They were individually housed in vials with 12 ml of the same well water, under the same 12 hr:12 hr dark:light cycle used in the mesocosm tank, and fed ad libitum with *C. vulgaris*. The algae used in the laboratory experiment were also grown in the same well water at room temperature and same light cycle. Because the well water did not contain enough phosphorous to promote algal growth, we added 20% of COMBO to all algal cultures (Kilham, Kreeger, Lynn, Goulden, & Herrera, 1998).

**Figure 1:**
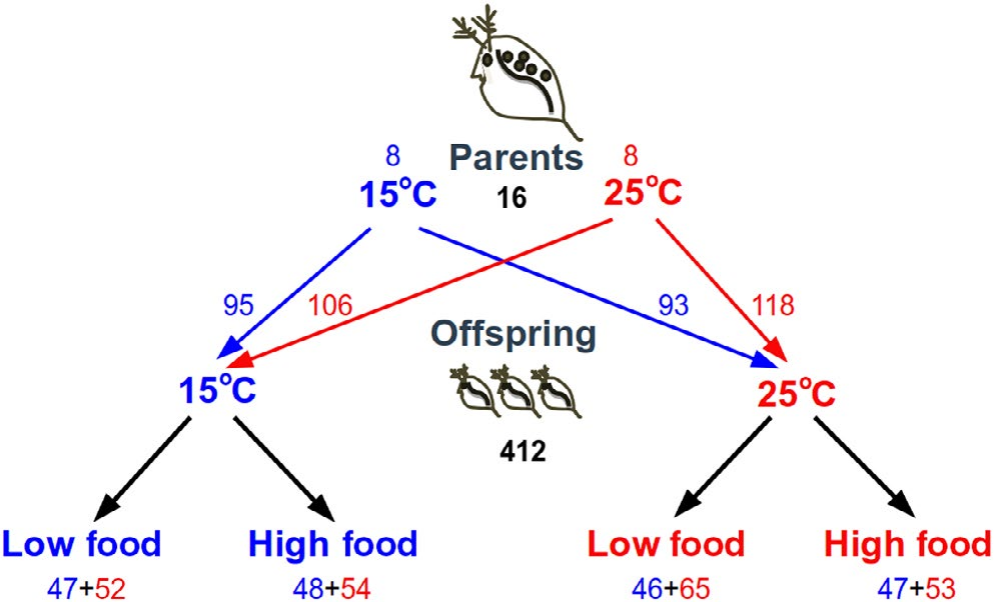
Schematic of the crossed design used to understand the effects of temperature on offspring size and life history. Numbers represent sample size (number of individuals) in each treatment

First generation offspring was used in the experiments only after each of the 16 females (eight in each temperature treatment) had produced three clutches under laboratory conditions. Females were checked for new offspring every morning. Within 24 hr, all individuals (~24 individuals per female; see Figure 1 for sample size in each treatment) were measured and haphazardly assigned to one of the four treatment combinations in a factorial design: high or low level of food abundance and high or low temperature. All offspring were individually housed in vials of the same size and medium used for the adults and placed in incubators, at either 15 or 25°C with a 12 hr dark:12 hr light cycle. We replaced 2/3 of the medium of all individuals daily to maintain constant algal concentration and replaced the whole vial once a week and whenever a female produced a new clutch. Food added was 6 × 10^5^ cells/ml for the high food treatment and 1 × 10^5^ cells/ml for the low food treatment. The high food level was chosen to allow females to have an excess of food after 24 hr (as determined from preliminary trials), and the low level was chosen to represent one half of this amount.

To measure body and clutch size, all individuals were photographed daily with digital camera attached to a dissecting microscope. Body size was measured from the anterior point of the eye to the base of the tail spine, and clutch size was estimated from the pictures as the number of eggs in the brood chamber.

To understand the effect of temperature and food abundance on adult body size and other demographic parameters, we used a robust mixed effect linear model (a form of weighted mixed effect model) to reduce the influence of potential outliers and, at the same time, to control for the potential influence of the individual mother and maternal effects (mother ID was entered as a random effect). To additionally control for maternal effects, we also included the temperature parents had been raised at as an explanatory variable. This was important, because the mothers we used could have experienced plastic changes caused by different levels of temperature they experienced in the mesocosm tanks. Body size, age and size at first reproduction, time between clutches, clutch size (mean of number of eggs produced), and lifetime reproductive success (total number of eggs produced) were used as response variables. The explanatory variables were temperature and food abundance (and their interaction) for body size, and temperature for all other life history parameters. We did not include food in the analysis of most life history parameters because individuals exposed to high temperature and low levels of food did not reproduce. For model inference, we used Wald confidence intervals (CI) and evaluated whether the parameter estimate fell within the 95% confidence limits for the Wald test. We investigated the effects of food and temperature on survival probability using a robust Cox proportional hazards model with the same explanatory variables as above. To facilitate residual normality, we log transformed all response variables prior to analysis. The regressions were fit with the *robustlmm* package (Koller, 2016), and the Cox model was fit with the *coxrobust* package (Bednarski & Borowicz, 2006). We also tested at what age the average size individuals at low temperature exceeded those from high temperature by comparing the mean body size for each day of the trial with a Welsh *t* test.

## 3 RESULTS

**Figure 2:**
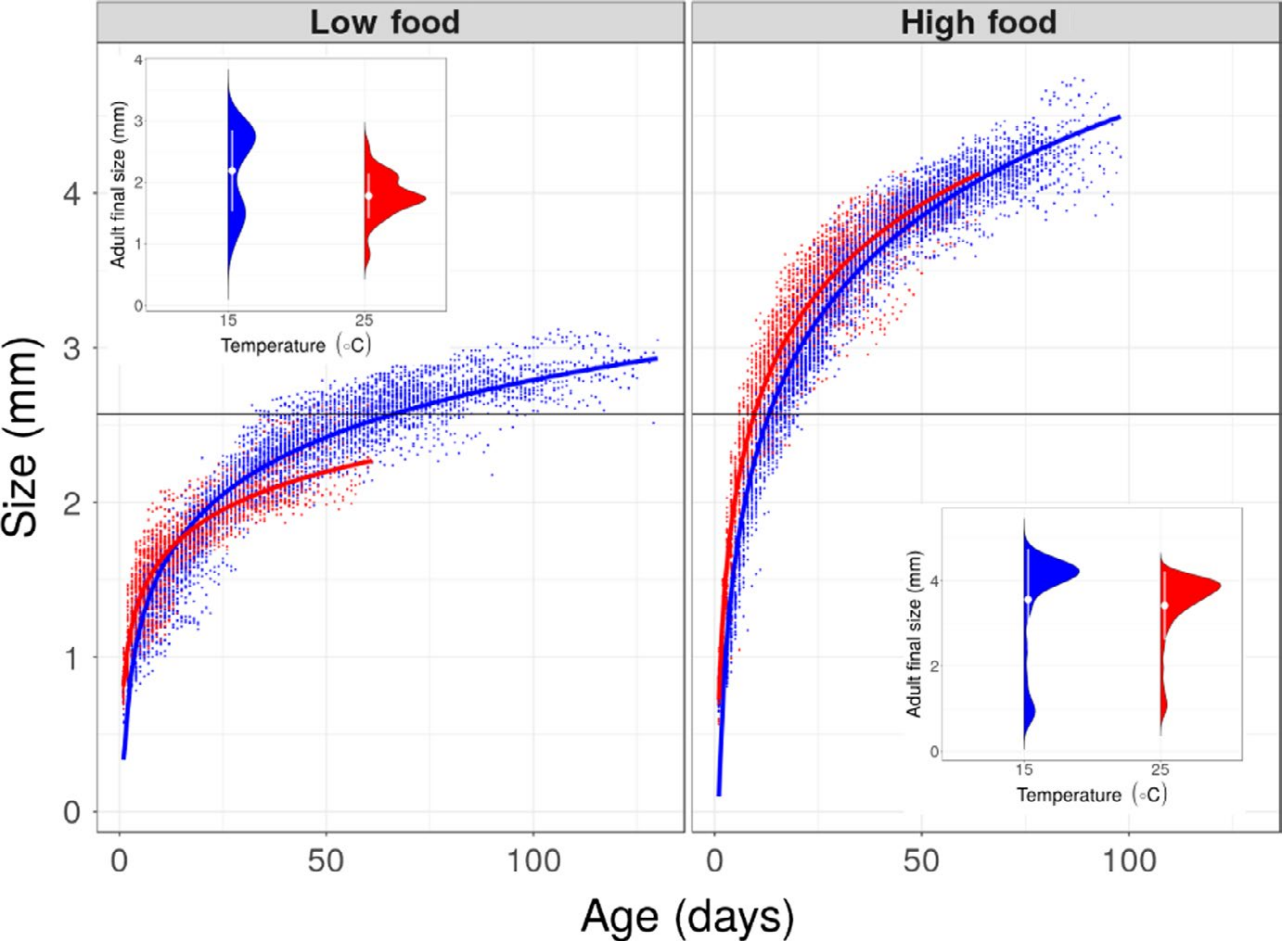
Body size of 412 *Daphnia magna* individuals, measured daily throughout their entire lives. Red and blue represent individuals exposed to high and low temperature, respectively. Horizontal lines represent average size of first reproduction for all treatment where at least one individual reproduced (~2.58 mm; size at first reproduction was not statistically different between treatments). Red and blue lines represent fitted values obtained by the equation *y* = *a* + *b* × log (*x*) where *y* is size, *x* is age, and *a* and *b* are parameters estimated from the data (see Table S3 for parameter values). Inset figures represent maximum body size of 412 *Daphnia magna* individuals. White points and lines beside the violin plots represent the mean ± 1 standard deviation

**Figure 3:**
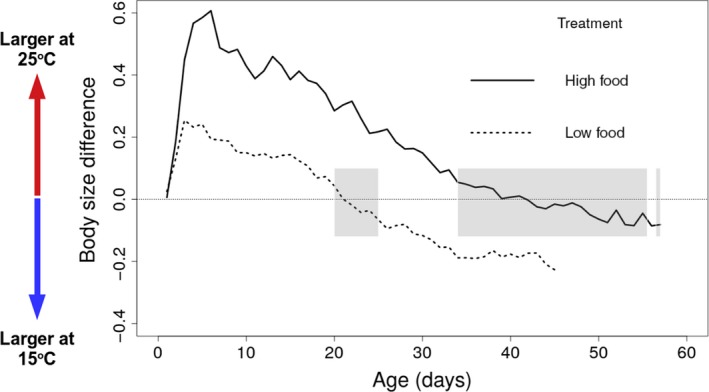
Difference in body size between temperature treatments, calculated as the mean body size of individuals exposed to high temperature minus the mean body size of individuals exposed to low temperature. We only compared means if treatments had at least 10 individuals. Positive values indicate that individuals at high temperature were larger than individuals at low temperature. Shaded gray areas indicated that there is no statistical difference between the daily mean body size at α = 0.05

**Figure 4:**
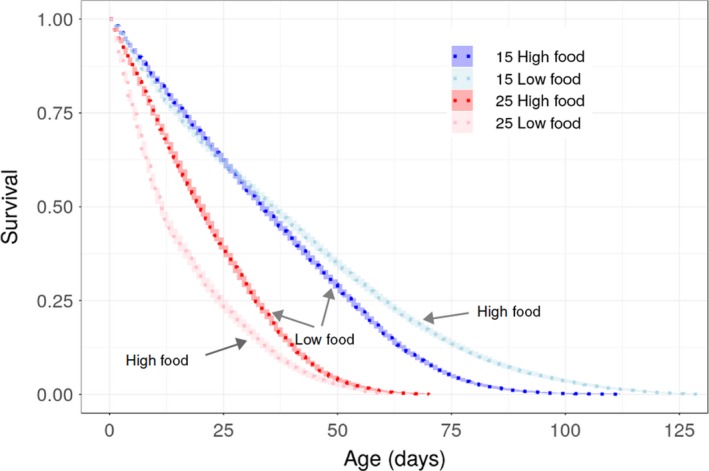
Survival probability of *Daphnia magna* individuals in high vs. low temperature and high vs. low food treatments, estimated using a Cox proportional hazards regression model. Shaded areas represent 95% confidence intervals

Experimentally imposed differences in food availability altered the magnitude of temperature‐dependent effects (Table 1 and Table S1, Figure 2 and Figure S1): under low food conditions, high temperature resulted in a reduction of 20% in maximum body size, compared with a reduction of 4% under high food conditions (Figure 2; significant interaction between temperature and food; β = 0.133, 95% Wald confidence interval = 0.042, 0.224; Table 1 and Table S2). Individuals exposed to low levels of food abundance were larger when raised at high temperature for about half of their life span (Figures 3 and 4). In contrast, well‐fed individuals raised at high temperature were larger than well‐fed individuals raised at low temperature for most of their lives (Figures 3 and 4). Food abundance also mediated the temperature‐dependent effect on survival probability: high levels of food abundance reduced survival probability in the low temperature treatment, whereas high levels of food abundance had the opposite effect at high temperature, resulting in increased survival probability (Figure 4; significant interaction between temperature and food; β = −0.558, SE = 0.035, *p* < .001; Table S3).

**Table 1:** Average and ± standard deviation of demographic parameters obtained for individuals exposed to one of treatments: low (15°C) and high temperature (25°C) and/or low and high food availability. There was no reproduction when individuals were exposed to both 25°C and low food treatment. Numbers are parenthesis in the header represent sample size for each treatment (*n* = 412). LTRS refers to lifetime reproductive success

	15°C		25°C	
	Low food (99)	High food (102)	Low food (111)	High food (100)
Adult body size (mm)	2.19 (±0.66)	3.55 (±1.19)	1.78 (±0.36)	3.42 (±0.80)
Life span (days)	51.82 (±37.86)	55.09 (±29.24)	19.09 (±15.38)	35.14 (±16.71)
Age at 1st reproduction (days)	57.7 (±11.90)	14.96 (±2.47)	‐	8.61 (±1.65)
Size at 1st reproduction (mm)	2.58 (±0.13)	2.60 (±0.19)	‐	2.58 (±0.19)
Time between clutches (days)	58.37 (±37.60)	6.43 (±3.40)	‐	3.54 (±1.10)
Clutch size (number of eggs)	1.34 (±0.41)	15.89 (±3.95)	‐	10.30 (±2.61)
LTRS (number of eggs)	3.32 (±2.34)	196.56 (±85.91)	‐	130.29 (±70.91)

**Figure 5:**
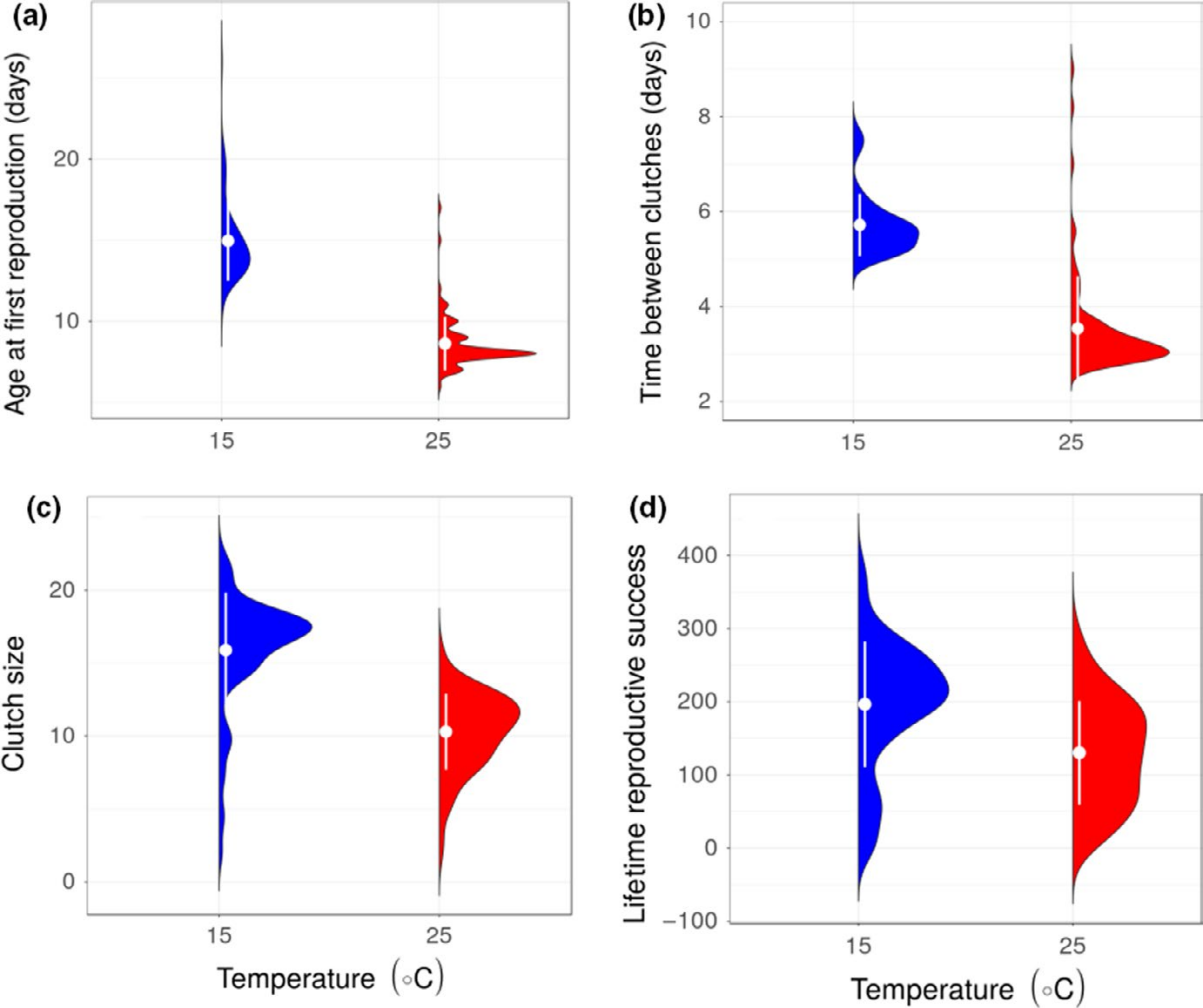
Distribution of age at first reproduction (a), time between clutches (b), mean clutch size (c), and lifetime reproductive success (d) for *Daphnia magna* individuals in the high food treatment. Only a few individuals reproduced in the low food treatment. White points and lines beside the violin plots represent the mean ± 1 standard deviation

High temperature resulted in a faster life cycle for individuals with high food abundance, with earlier age at first reproduction (β = −0.548, CI = −0.585, −0.511; Figure 5a, Table 1 and Table S2) and shorter time between clutches (β = −0.553, CI = −0.588, −0.517; Figure 5b, Table 1 and Table S2). This “live fast, die young” life history response, however, came at the cost of reduction in average clutch size (β = −0.453, CI = −0.514, −0.393; Figure 5c, Table 1 and Table S2) and diminished lifetime reproductive success (β = −0.469, CI = −0.658, −0.280; Figure 5d, Table 1 and Table S2). This cost was most extreme in the treatment combining low food with high temperature, in which no individuals reproduced. Size at first reproduction (~2.58 mm) was the same for all treatments where females produced at least one clutch (there was no effect of temperature on size at first reproduction; β = −0.009, CI = −0.062, 0.044; Figure 2 Table 1 and Table S2).

## 4 DISCUSSION

Our experimental results show that the shrinking effect caused by high temperature in *Daphnia* was food‐dependent, indicating that high levels of food intake can partially compensate for temperature‐dependent constraints on body size. Moreover, accelerated life history responses were observed only under high levels of food abundance. At low levels of food, individuals did not reproduce when exposed to high temperature, even though their life span was similar to those individuals exposed to high temperature and high food concentration. This indicates that the fast life cycle due to warming was most pronounced when energy intake exceeded the amount required to meet metabolic demand. This could have important consequences for the resilience of ecosystems, because fast life cycles are hypothesized to promote rapid rates of population growth, and hence instability (Bestion et al., 2015). On the other hand, the combination of high temperature and low levels of food abundance, leading to compromised reproduction, would obviously have a negative impact on population growth rate. Consequently, populations exposed to extended periods of both high temperature and low levels of food abundance could be particularly vulnerable to population collapse and perhaps even extinction.

Size at first reproduction was the same for all treatments where individuals were able to reproduce. Even at low temperature, *Daphnia* individuals had an extended life span and many of them achieved the minimal size to reproduce, but a few were able to produce a clutch. This suggests a relocation of resources toward growth that compromises reproduction. In many species, including *Daphnia magna* (Ebert, 1992), individuals must achieve a threshold size to reproduce. When exposed to low food conditions, some individuals might relocate resources toward growth at the cost of reproduction. Finding enough resources for maintenance and reproduction is a common challenge for many species during the breeding season. A combination of high ambient temperature due to global warming and low food abundance, caused for example by mismatches between resources and consumers (Both et al., 2010; Cahill et al., 2012), could be particularly lethal. It could even create an ecological trap because of the dissociation between the reliable cue to invest in reproduction under warming seasonal conditions and the fitness costs that this strategy would result under low levels of food abundance (Schlaepfer, Runge, & Sherman, 2002).

The mechanisms linked to body size changes at different temperatures may have differed between high and low food abundance treatments. At high levels of food availability, individuals raised under high temperature conditions tended to be larger for most of their lives compared with individuals raised under cooler conditions. Nonetheless, individuals raised at cooler temperatures, regardless of their food levels, tended to reach larger size before they died due to their extended life span. We speculate that this could be due to the high metabolic costs of maintaining an accelerated life cycle. At low food levels, growth patterns resembled those expected for the temperature‐size rule, that is, fast growth at young ages, with a lower final body size than those observed at low temperature (Atkinson, 1996; Audzijonyte et al., 2017). Oxygen limitation has been hypothesized as a cause of the temperature‐dependent reduction in ectotherm body size (Atkinson, Morley, & Hughes, 2006). However, the oxygen hypothesis leads to the prediction that organisms relying on diffusive uptake should be particularly sensitive to temperature stress, regardless of food abundance (Rollinson & Rowe, 2018), a pattern that we did not observe in our experiments. Although we did not measure oxygen intake, our results suggest that changes in body size distribution are influenced by a complex mix of responses in growth, reproduction, and survival. System‐specific variation in the relative degree of response in each of these variables might help to explain some of the conflicting results seen in the published literature on the effect of warming on mean adult body size (Atkinson et al., 2006; Audzijonyte et al., 2017; Kooijman & Kooijman, 2010; Rollinson & Rowe, 2018; Vidal, 1980).

## Conflict of Interest

None declared.

## AUTHOR CONTRIBUTIONS

GSB conceived the idea with JMF; GSB ran the experiments and collected the data assisted by XW; GSB, TA, and MG analyzed the data; GSB wrote the first draft of the manuscript. All authors discussed the ideas and commented on subsequent drafts of the manuscript.

## Data Availability

The data are available at the Figshare Repository https://10.6084/m9.figshare.11116640.
