# Supplementary figures and images for: Food availability modulates temperature‐dependent effects on growth, reproduction, and survival in Daphnia magna
Source: Ecol Evol. 2019 Dec 27;10(2):756–62. doi: 10.1002/ece3.5925 (PMC6988562; doi:10.1002/ece3.5925)

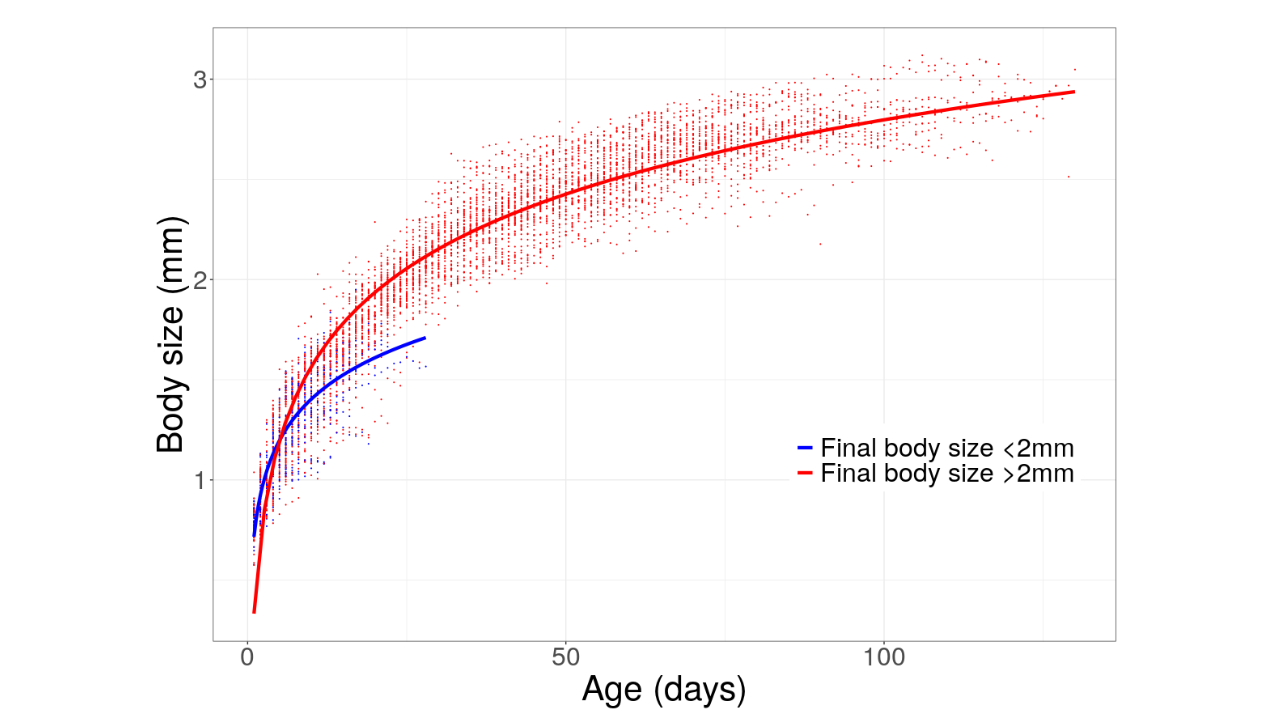

Supplement: Supplementary file 1 [file ECE3-10-756-s001.tiff]
