# Supplementary material for: Food availability modulates temperature‐dependent effects on growth, reproduction, and survival in Daphnia magna
Source: Ecol Evol. 2019 Dec 27;10(2):756–62. doi: 10.1002/ece3.5925 (PMC6988562; doi:10.1002/ece3.5925)
Supplement: Supplementary file 2 [file ECE3-10-756-s002.docx]

**Appendix**

**Table S1.** Parameter estimates for robust linear mixed effect models used to investigate the effects of temperature on a number of life history parameters.

| **Parameter** | **Estimate** | **2.5%** | **97.5%** | **Variance** | **SE** |
| --- | --- | --- | --- | --- | --- |
| **Adult Body Size** | | | | |  |
| Mother ID |  |  |  | 0.001 | 0.034 |
| Residual |  |  |  | 0.052 | 0.229 |
| Intercept | 0.796 | 0.739 | 0.854 |  |  |
| High Food | 0.547 | 0.482 | 0.612 |  |  |
| High Offspring Temperature | -0.210 | -0.274 | -0.146 |  |  |
| High Parent Temperature | -0.023 | -0.080 | 0.034 |  |  |
| High Food:High Offspring Temperature | 0.133 | 0.042 | 0.224 |  |  |
| **Age at 1^st^ reproduction** | | | | |  |
| Mother ID |  |  |  | 0.000 | 0.000 |
| Residual |  |  |  | 0.013 | 0.115 |
| Intercept | 2.757 | 2.722 | 2.792 |  |  |
| High Offspring Temperature | -0.548 | -0.586 | -0.511 |  |  |
| High Parent Temperature | -0.134 | -0.172 | -0.095 |  |  |
| **Time between clutches** |  |  |  |  |  |
| Mother ID |  |  |  | 0.001 | 0.028 |
| Residual |  |  |  | 0.012 | 0.108 |
| Intercept | 1.756 | 1.716 | 1.797 |  |  |
| High Offspring Temperature | -0.553 | -0.588 | -0.517 |  |  |
| High Parent Temperature | -0.038 | -0.085 | 0.009 |  |  |
| **Clutch size** |  |  |  |  |  |
| Mother ID |  |  |  | 0.006 | 0.077 |
| Residual |  |  |  | 0.033 | 0.182 |
| Intercept | 2.897 | 2.813 | 2.981 |  |  |
| High Offspring Temperature | -0.453 | -0.514 | -0.393 |  |  |
| High Parent Temperature | -0.183 | -0.286 | -0.080 |  |  |
| **Life time reproductive success** |  |  |  |  |  |
| Mother ID |  |  |  | 0.000 | 0.000 |
| Residual |  |  |  | 0.334 | 0.578 |
| Intercept | 5.276 | 5.100 | 5.451 |  |  |
| High Offspring Temperature | -0.469 | -0.658 | -0.279 |  |  |
| High Parent Temperature | -0.059 | -0.252 | 0.134 |  |  |

**Table S2.** Parameter estimates for the mixed effect Cox proportional hazards regression model to investigate the effects of temperature and food abundance on survival.

| **Parameter** | **Coefficient** | **SE** | **p** |
| --- | --- | --- | --- |
| High Food | 0.253 | 0.021 | <0.001 |
| High Offspring Temperature | 1.316 | 0.028 | <0.001 |
| High Parent Temperature | -0.141 | 0.070 | 0.045 |
| High Food:High Offspring Temperature | -0.558 | 0.035 | <0.001 |

**Table S3.** Parameter estimates from equation$y=a+b*log\left( x \right)$used in figure 1 in the main text.

| **Treatment** | **Estimate** | **SE** | **t-value** | **P** |
| --- | --- | --- | --- | --- |
| **15^o^C Low Food** |  |  |  |  |
| a | 0.354 | 0.008 | 45.08 | <0.001 |
| b | 0.527 | 0.002 | 231.98 | <0.001 |
| **15^o^C High Food** |  |  |  |  |
| a | 0.105 | 0.010 | 10.76 | <0.001 |
| b | 0.953 | 0.003 | 330.42 | <0.001 |
| **25^o^C Low Food** |  |  |  |  |
| a | 0.811 | 0.009 | 93.14 | <0.001 |
| b | 0.355 | 0.003 | 104.56 | <0.001 |
| **25^o^C High Food** |  |  |  |  |
| a | 0.698 | 0.011 | 62.08 | <0.001 |
| b | 0.83 | 0.004 | 214.40 | <0.001 |

**Figure S1.** Growth curve for individuals at low temperature and low food abundance that achieved body size > or < 2mm. The apparent binomial distribution (see figure 2 in the main text) could be caused by individuals that adopted two different strategies. One strategy is to invest more in reproduction, resulting in less energy for growth and maintenance. The other strategy is to invest more in growth and longer life span. Under low food levels, individuals that invest more in reproduction might not have enough energy for growth and maintenance and might die earlier (blue dots/line in the figure below) than individuals that invest more in growth and delay reproduction (red dots/line).
